# Retrospective study on the therapeutic efficacy of zinc acetate hydrate administration to patients with hypozincemia-induced dysgeusia

**DOI:** 10.1186/s12903-023-02866-7

**Published:** 2023-03-18

**Authors:** Tomoaki Shintani, Kouji Ohta, Toshinori Ando, Yasutaka Hayashido, Souichi Yanamoto, Mikihito Kajiya, Hideki Shiba

**Affiliations:** 1 Center of Oral Clinical Examination, Hiroshima University Hospital, Hiroshima, Japan; 2 Department of Public Oral Health, Graduate School of Biomedical and Health Sciences, Hiroshima University, Hiroshima, Japan; 3 Department of Oral Oncology, Graduate School of Biomedical and Health Sciences, Hiroshima University, Hiroshima, Japan; 4 Department of Biological Endodontics, Graduate School of Biomedical and Health Sciences, Hiroshima University, Hiroshima, Japan

**Keywords:** Dysgeusia, Hypozincemia, Copper/zinc ratio, Zinc acetate hydrate, Hypocupremia

## Abstract

**Background:**

Dysgeusia is a relatively early symptom of zinc deficiency, and zinc replacement is effective in treating dysgeusia. The administration of zinc acetate hydrate (ZAH) was approved in 2017 for patients with hypozincemia in Japan. This retrospective study was conducted to explore the efficacy and safety of ZAH administration in patients with hypozincemia-induced dysgeusia.

**Methods:**

Patients with hypozincemia-induced dysgeusia who visited our hospital from May 2013 to December 2019 were included in this study. ZAH (zinc content; 50 mg/day) was administered to 42 patients for 24 weeks. The taste test was performed using the filter paper disk method, and the total cognitive thresholds of the left and right chorda tympani regions were used. Changes in taste function, serum zinc and copper levels, and copper/zinc ratio were analyzed. A total of 28 patients who received polaprezinc (PPZ, zinc content; 34 mg/day) for 24 weeks, who were prescribed until ZAH was approved, were registered as controls.

**Results:**

Serum zinc levels at 12 and 24 weeks after ZAH or PPZ administration were higher than those before administration. These levels were significantly higher in the ZAH-treated group than in the PPZ-treated group. However, serum copper levels did not significantly change before and after administration. In the taste test, the taste thresholds for the acidity and salty at 12 and 24 weeks after ZAH administration were significantly decreased compared to before administration. In contrast, in the PPZ group, the taste thresholds for the acidity and salty were significantly decreased 24 weeks after administration.

**Conclusions:**

ZAH (50 mg/day) administration was effective in improving the gustatory sensitivity of patients with dysgeusia and hypozincemia 12 weeks after administration without affecting the serum copper level. ZAH was also more effective than PPZ.

## Background

Zinc (Zn), an essential trace element, is related to enzymatic activities and exerts several physiological roles in the body [1–3]. Zn serves as a cofactor in DNA polymerase and in Zn-finger proteins that are addicted to DNA and regulates protein synthesis [4, 5]. Zn deficiency is diagnosed on the basis of clinical symptoms due to lower serum Zn levels [6]. The symptoms of Zn deficiency include loss of appetite, stunting, skin symptoms, hair loss, gonadal dysfunction, dysgeusia, delayed wound healing, and immune decline [7]. Moreover, previous studies have reported Zn deficiency in diseases such as hepatitis C, liver cirrhosis, diabetes, inflammatory bowel disease, and chronic renal diseases [8, 9]. Other studies have also shown that Zn deficiency elevates the concentration of inflammatory cytokines and oxidative stress and induces the apoptosis of endothelial cells [10–12]. Zn supplementation was found to decrease the concentrations of plasma C-reactive protein and interleukin (IL)-6 in elderly subjects [13]. Consequently, Zn replacement therapy is recommended as a treatment for patients with Zn deficiency [14].

In recent years, the number of patients visiting a hospital with a primary complaint of dysgeusia has been increasing annually in Japan [15]. The Japanese survey conducted in 2003 reported that the number of patients with dysgeusia was 240,000 per year [16]. The causes of dysgeusia include drugs and stress, but the most common cause is Zn deficiency [17, 18]. Particularly, Zn is present in the squamous epithelium containing the taste buds of the tongue papilla in a high concentration, and the taste buds contain several Zn enzymes such as alkaline phosphatase, acid phosphatase, and cyclic AMP phosphodiesterase [19]. Kinomoto et al. reported about the closure of the parakeratinized epithelium in the taste buds of the fungiform papillae and the circumvallate papillae in rats with Zn deficiency [20]. The Zn supplementation polaprezinc (PPZ), which has been approved in Japan as an antiulcer agent, had a therapeutic effect in patients with idiopathic taste disorders, including those with hypozincemia [19]. Animal experiments showed that the administered PPZ was distributed to the lingual epithelium and restored Zn concentration in Zn-deficient rats, resulting in improvement of the proliferation of taste bud cells [21]. To date, no Zn drugs for the treatment of Zn deficiency have been approved as a therapeutic agent. In 2017, the administration of zinc acetate hydrate (ZAH), which contains a higher amount of Zn than that in PPZ and is used for the treatment of rare inherited disorder Wilson’s disease that causes copper (Cu) accumulation in vital organs, was approved for treating patients with hypozincemia in Japan [22, 23]. ZAH has demonstrated efficacy in patients with cirrhosis, in which Zn deficiency is common [22]. A recent study has shown that ZAH is more effective than PPZ in elevating serum Zn levels in patients under maintenance hemodialysis [24]. It has also been shown that ZAH administration to patients with inflammatory bowel disease with Zn deficiency may normalize serum Zn levels and improve disease activity, particularly in patients with Crohn’s disease [25].

Zn and Cu, which are divalent metal cations, are known to antagonize the absorption of each other in the intestinal tract [26]. Thus, Zn supplementation can easily cause a decrease in serum Cu concentration. In fact, it has been reported that pancytopenia due to hypocupremia was observed in three patients under maintenance hemodialysis with hypozincemia who received ZAH for 4–7 months [27]. Another study also reported that serum Cu levels were significantly decreased at 3 months after PPZ administration in patients under hemodialysis [24]. Nevertheless, one study showed that there is no relationship between serum Zn concentration and dysgeusia and that the Cu/Zn ratio is a more important diagnostic marker for dysgeusia [28].

Reports on the administration of ZAH to patients with Zn-deficient dysgeusia are insufficient. Thus, in this study, we retrospectively investigated the effects of ZAH administrations on taste and changes in serum Zn and Cu levels and Cu/Zn ratio in patients with hypozincemia-induced dysgeusia. Patients who received PPZ for the same period were used as controls. Furthermore, patients who received ZAH were divided into the improved and non-improved groups on the basis of the results of the taste test, and their clinical characteristics were compared.

## Methods

### Subjects

In total, 70 outpatients who visited the study hospital with a complaint of taste disorder between May 2013 and February 2021, with zinc deficiency (serum zinc level < 80 µg / dL) and a total taste test score of 28 or higher on the filter paper disc method shown below were diagnosed with hypozincemia-induced dysgeusia and were included in this study. Of these 70 patients, 42 received 50 mg (element Zn)/day of ZAH (Nobelzin® tablet; Nobelpharma Co., Ltd., Tokyo, Japan) and were treated daily for 24 weeks. The remaining 28 patients received 150 mg/day of PPZ (Promac® granules; Zeria Pharmaceutical Co., Ltd., Tokyo, Japan), which contained approximately 34 mg/day of Zn, and were treated daily for 24 weeks. Both drugs were taken orally after breakfast and dinner. PPZ was prescribed to patients with dysgeusia and hypozincemia before April 2017. Following its approval for treatment in Japan in April 2017, ZAH, but not PPZ, was prescribed to patients.

### Paper filter disk method

The paper filter disk method was performed to test the recognition of the four flavors sweet, salty, sour, and bitter using the kit (Taste Disc®; Sanwa Chemistry Institute, Nagoya, Japan) [29]. Gustatory sensitivity was investigated for two regions of the bilateral location in the chorda tympani nerve. Unless the patient recognized the taste, the test was continued with solutions of higher concentrations until the correct response was given. A scoring system was used, ranging from 1 to 6, where scores 1 and 5 represent the lowest and highest measurable thresholds, respectively. Score 6 indicates that no flavor was experienced even at the highest concentration. The sum of the scores on both sides for each flavor was taken as a total taste score. The paper filter disk method was conducted at the first visit and 12 and 24 weeks after the drug administration. Patients whose total taste scores at 12 and 24 weeks after the administration of the Zn preparation were lower than those before its administration, i.e., at the first visit, were defined as those who exhibited improvement (improved group). Patients whose total taste scores after the administration of Zn preparation were higher or unchanged compared to that before its administration were defined as those who did not exhibit improvement (non-improved group). All taste tests were performed by the same dentist at the outpatient dentistry clinic.

### Measurement of serum Zn and Cu levels

All patients attended the clinic in the morning due to the circadian rhythm of serum zinc levels. Serum Zn and Cu levels were measured by colorimetry (Zn in our laboratory, Cu; LSI Medience Corporation, Tokyo, Japan). The reference values for serum Zn and Cu were 80–130 and 68–128 µg/dL, respectively [30, 31].

### Cu/Zn ratio

The Cu/Zn ratio was examined because Yanagisawa et al. reported that this ratio was a useful diagnostic marker for taste disorders, and a value of 1.1 may be a threshold level for detecting taste disorders [28].

### Statistical analysis

Statistical analysis was conducted using BellCurve for Excel (Social Survey Research Information Co., Ltd., Tokyo, Japan). Data were expressed as mean ± SD (standard deviation). For continuous variables, changes in Zn, Cu, Cu/Zn ratio and taste over time were compared using paired *t* test, whereas other comparisons were performed using two-sample *t* test. The χ^2^ test was used for analyzing categorical variables. In all analyses, *P* values of < 0.05 were considered to be statistically significant.

### Ethical considerations

We obtained approval for this study from the research ethics board of Hiroshima University (approval number: epidemiology—1485). The study protocol was posted on the websites. Patients opted out from the study if they did not wish to give consent. The informed consent was waived.

## Results

### Subject characteristics

Seventy patients diagnosed with taste disorders with hypozincemia entered into this study. Table 1 summarizes the baseline characteristics of these patients. The ZAH/PPZ group consisted of 14 men (33%)/28 women (67%) or 13 men (46%)/15 women (54%), and the mean age of subjects in the ZAH/PPZ group was 73.2/68.4 years (range, 61–88/55–98 years), respectively. The mean body weight of subjects in the ZAH/PPZ group was 63.9/65.9 kg (range, 58.4–75.5/53.8–78.1 kg). The mean serum Zn level was 68.3/73.2 µg/dL (range, 58–76/63–78 µg/dL), and the mean serum Cu level was 102.7/108.7 µg/dL (range, 72–130/76–135 µg/dL) in the ZAH/PPZ group, respectively. The mean Cu/Zn ratio in the ZAH group was 1.50 (range, 1.0–1.8), and that in the PPZ group was 1.62 (range, 0.9–1.9). In the ZAH group, there were 35 patients (83%) with a Cu/Zn ratio of ≥ 1.1, whereas in the PPZ group, there were 19 patients (73%). The taste scores (ZAH group vs PPZ group) were 9.3 ± 1.8 vs 9.4 ± 2.1 for sweetness, 9.3 ± 1.9 vs 9.3 ± 2.5 for saltiness, 10.0 ± 2.3 vs 10.2 ± 2.0 for acidity, 9.4 ± 2.2 vs 10.0 ± 1.8 for bitterness, and 37.9 ± 5.2 vs 38.8 ± 4.6 for total. Thus, no significant differences were observed between the ZAH and PPZ groups.

**Table 1:** Baseline characteristics of subjects

Parameter	ZAH (*n* = 42)	PPZ (*n* = 28)	*P* value
**Sex (M / F)**	**14 / 28**	**13 / 15**	**0.139**^**a**^
**Age (years)**	**73.2 ± 11.3**	**68.4 ± 12.5**	**0.188**^**b**^
**Body weight (kg)**	**63.93 ± 13.74**	**65.92 ± 11.21**	**0.276**^**b**^
**Zn (µg / dL)**^**c**^	**68.3 ± 8.7**	**73.2 ± 10.0**	**0.109**^**b**^
**Cu (µg / dL)**^**d**^	**102.7 ± 29.4**	**108.7 ± 29.4**	**0.234**^**b**^
**Cu / Zn ratio**	**1.50 ± 0.48**	**1.62 ± 0.76**	**0.271**^**b**^
**Cu / Zn ratio (< 1.1 / ≧1.1)**	**7 / 35**	**7 / 19**^**e**^	**0.36**^**a**^
**Taste scores**^**f**^			
** Sweet**	**9.3 ± 1.8**	**9.4 ± 2.1**	**0.96**^**b**^
** Salty**	**9.3 ± 1.9**	**9.3 ± 2.5**	**0.84**^**b**^
** Sour**	**10.0 ± 2.3**	**10.2 ± 2.0**	**0.96**^**b**^
** Bitter**	**9.4 ± 2.2**	**10.0 ± 1.8**	**0.2**^**b**^
** Total**^**g**^	**37.9 ± 5.2**	**38.8 ± 4.6**	**0.5**^**b**^

Values are expressed as means ± standard deviation

*Zn* zinc, *Cu* copper, *ZAH* zinc acetate hydrate, *PPZ* Polaprezinc

^a^ Test used for analysis: *X*^2^ test

^b^ Test used for analysis: two-sample *t* test

^c^ The reference value is 80–130 µg/dL

^d^ The reference value is 68–128 µg/dL

^e^ 2 patients have no Cu data

^f^ The scores of the bilateral location in the chorda tympani nerve using the paper filter disk method

^g^ Sum of the above 4 flavors

### Changes in serum Zn and Cu levels

**Fig. 1:**
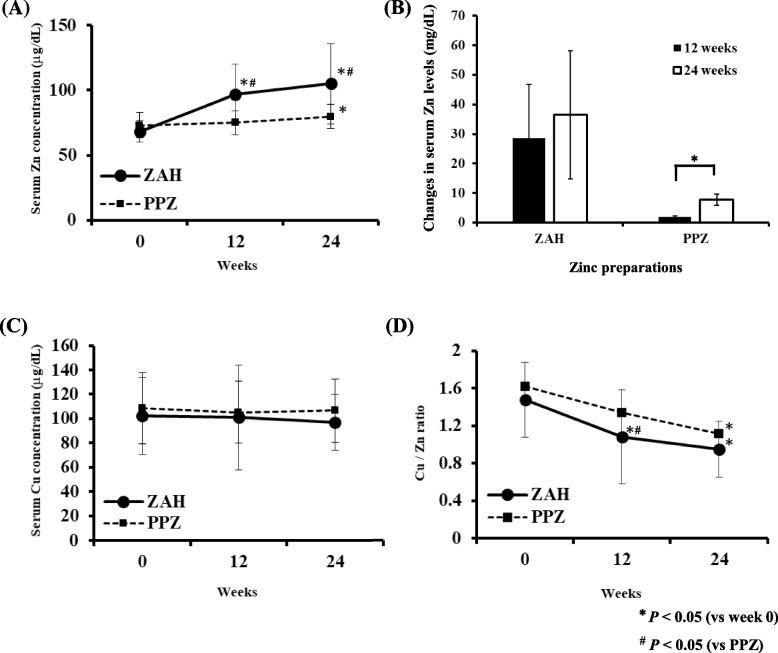
Changes in serum Zn, Cu levels and Cu/Zn ratio in patients receiving ZAH or PPZ. Serum Zn levels increased after receiving oral Zn supplements. These levels were significantly higher in the ZAH-treated group than in the PPZ-treated group (**A**). The mean change in serum zinc levels was greater in the group that received the zinc supplements for 24 weeks than in the group that received it for 12 weeks (**B**). No change was observed in serum Cu levels after the oral administration of Zn supplements (**C**). After the administration, the Cu/Zn ratio was lower in both groups than that before the administration (**D**). Each bar represents mean ± standard deviation. ^*^P < 0.05 (vs week 0, paired *t* test)

The changes in the concentrations of serum Zn and Cu after drug administration in both groups are shown in Fig. 1A, B and C. The serum Zn levels in the ZAH group significantly increased at 12 and 24 weeks after drug administration (*P* < 0.05, Fig. 1A), whereas those in the PPZ group significantly increased only at 24 weeks (*P* < 0.05, Fig. 1A) although the observed increase was very low. In terms of the change in serum zinc levels before and after administration of zinc preparations, there was a significant increase in the group that received PPZ for 24 weeks compared to the group for 12 weeks (*P* < 0.05, Fig. 1B). The serum Zn levels at weeks 12 and 24 were significantly higher in the ZAH group than in the PPZ group (*P* < 0.05, Fig. 1A). The serum Cu levels showed no significant differences between before and after the administration of Zn supplements (Fig. 1C).

### Changes in the Cu/Zn ratio

The Cu/Zn ratio was significantly lower at 12 and 24 weeks after ZAH administration than that before its administration (*P* < 0.05, Fig. 1D). In contrast, the Cu/Zn ratio was significantly lower at 24 weeks after PPZ administration than that before its administration (*P* < 0.05, Fig. 1D).

### Evaluation of taste

**Fig. 2:**
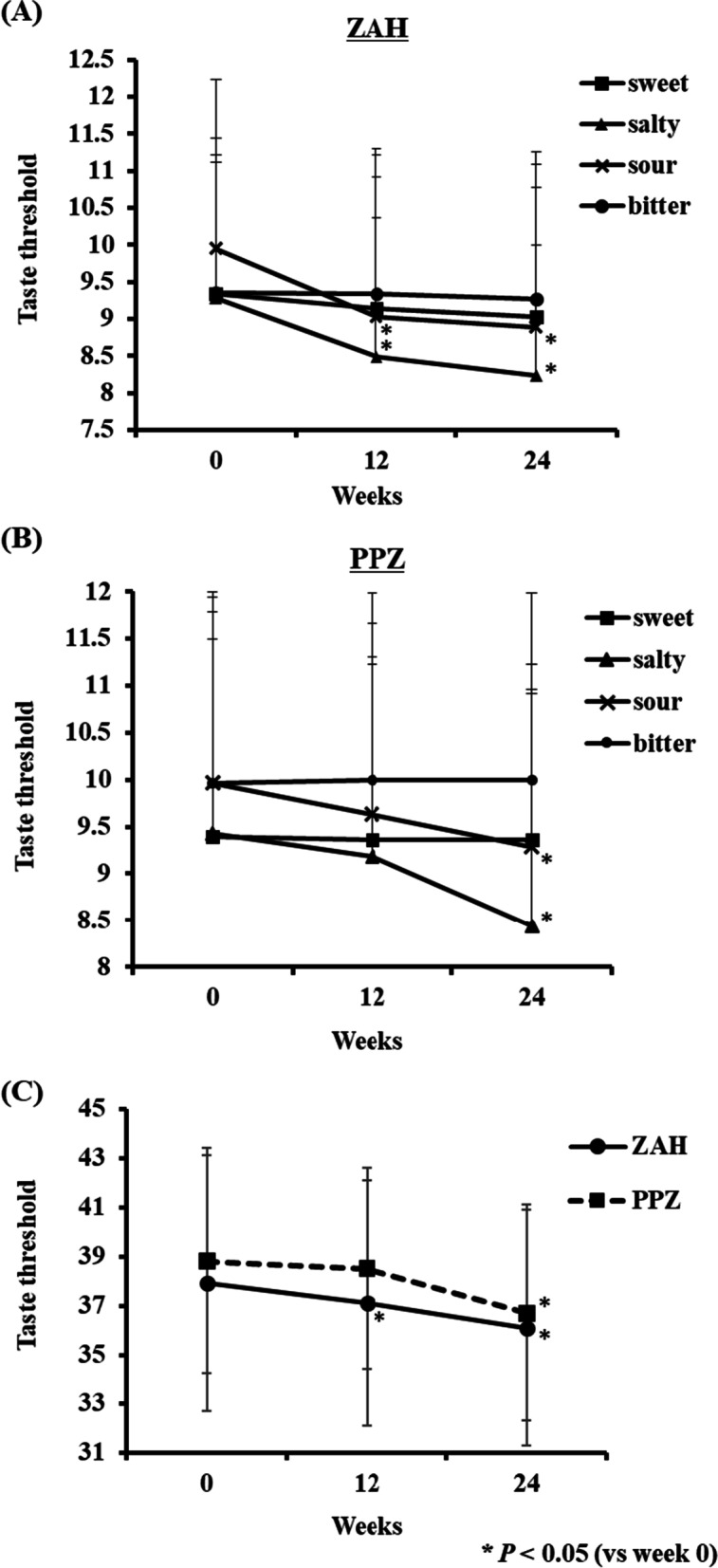
Changes in taste threshold in patients receiving ZAH or PPZ. At 12 and 24 weeks after ZAH administration, the taste scores of acidity and salty were significantly lower than those before administration (**A**). At 12 weeks after PPZ administration, the taste scores of acidity and salty were significantly lower than those before administration (**B**). In total of 4 tastes (sweet, salty, sour and bitter), a significant decrease in taste threshold was observed in the ZAH-administered group at 12 and 24 weeks after administration, and in the PPZ-administered group at 24 weeks after administration (**C**). Each bar represents mean ± standard deviation. ^*^ *P* < 0.05 (vs week 0, paired *t* test)

The taste threshold was examined using the paper filter disk method at 12 and 24 weeks after drug administration to evaluate whether gustatory sensitivity had improved compared to the first visit. In the ZAH group, the taste thresholds for the acidity and salty at 12 and 24 weeks after administration were significantly decreased compared to before administration (*P* < 0.05, Fig. 2A). In contrast, in the PPZ group, the taste thresholds for the acidity and salty were significantly decreased 24 weeks after administration (*P* < 0.05, Fig. 2B). In the total taste thresholds for the four tastes (sweet, salty, sour and bitter), the ZAH-administered group showed a significant improvement at 12 and 24 weeks after the administration compared to before administration, but the PPZ-administered group showed a significant improvement only at the 24 weeks (*P* < 0.05, Fig. 2C).

### Clinical characteristics of ZAH-treated patients

We next compared the clinical parameters of subjects in the improved and non-improved groups 24 weeks after ZAH administration (Table 2). The patients in the non-improved group were significantly younger than those in the non-improved group (age, 66.8 ± 11.2 vs 75.0 ± 11.8 years, *P* < 0.05). The serum Zn levels were significantly higher in the improved group than in the non-improved group (106.9 ± 9.0 vs 84.3 ± 7.8 µg/dL, *P* < 0.05). There was no difference in the Cu/Zn ratio between the two groups. However, the number of patients with a Cu/Zn ratio of < 1.1 was significantly higher in the improved group than in the non-improved group (23/26 vs 9/16, *P* < 0.05). Other clinical parameters did not differ between the two groups.

**Table 2:** Clinical characteristics of patients receiving ZAH at 24 weeks

Parameter	Improved^a^ (*n* = 26)	Non-improved^b^ (*n* = 16)	*P* value
**Sex (M / F)**	**10 / 16**	**5 / 11**	**0.75**^**c**^
**Age (years)**	**66.8 ± 11.2**	**75.0 ± 11.8**	** < 0.05**^**d**^
**Zn (µg / dL)**^**e**^	**106.9 ± 9.0**	**70.3 ± 7.8**	**0.109**^**d**^
**Cu (µg / dL)**^**f**^	**104.7 ± 37.1**	**101.3 ± 25.5**	**0.87**^**d**^
**Cu / Zn ratio**	**1.02 ± 0.25**	**1.32 ± 0.51**	**0.161**^**d**^
**Cu / Zn ratio (< 1.1 / ≧1.1)**	**23 / 3**	**9 / 7**	** < 0.05**^**d**^
**White blood cell (/mm**^**3**^**)**	**5610 ± 1278**	**6206** **±** **1894**	**0.54**^**d**^
**Red blood cell (× 10**^**4**^**/mm**^**3**^**)**	**489 ± 97**	**501 ± 87**	**0.82**^**d**^
**Hemoglobin (g/dL)**	**13.2 ± 3.9**	**13.5 ± 3.3**	**0.58**^**d**^
**AST (IU/L)**	**26.0 ± 9.7**	**23.4 ± 6.6**	**0.35**^**d**^
**ALT (IU/L)**	**27.3 ± 10.7**	**24.1 ± 10.4**	**0.34**^**d**^
**Albumin (g/dL)**	**4.3 ± 0.6**	**4.2 ± 0.7**	**0.72**^**d**^
**Creatinine (mg/dL)**	**0.73 ± 0.1**	**0.74 ± 0.1**	**0.65**^**d**^
**BUN (mg/dL)**	**12.6 ± 4.6**	**12.5 ± 2.5**	**0.97**^**d**^
**Triglyceride (mg/dL)**	**173.9 ± 115.3**	**204.9 ± 165.6**	**0.52**^**d**^

Values are expressed as means ± standard deviation

*Zn* zinc, *Cu* copper, *ZAH* zinc acetate hydrate

^a^ Patients whose total taste scores at 12 or 24 weeks after the administration of the Zn preparation were lower than those before its administration, were defined as improved group

^b^ Patients whose total taste scores after the administration of Zn preparation were higher or unchanged compared to that before its administration were defined as non-improved group

^c^ Test used for analysis: *X*^2^ test

^d^ Test used for analysis: two-sample *t* test

^e^ The reference value is 80–130 µg/dL

^f^ The reference value is 68–128 µg/dL

### Adverse events of ZAH administration

Approximately 24 weeks after ZAH administration, two patients complained of gastric discomfort, which disappeared without additional intervention after the completion of treatment. By contrast, there were no adverse events in the PPZ group.

## Discussion

We investigated the efficacy of ZAH administration in patients with hypozincemia-induced dysgeusia in comparison with PPZ administration. Moreover, we examined the relationship between Cu/Zn ratio and taste test results to confirm the usefulness of the Cu/Zn ratio as a diagnostic marker for dysgeusia.

Serum Zn levels were significantly higher in ZAH-treated patients 12 weeks after administration than those before administration. However, in PPZ-treated patients, these levels were significantly higher 24 weeks after the administration. Because the amount of Zn contained in ZAH was 50 mg/day, which was higher than that in PPZ (34 mg/day), the serum Zn level in patients taking ZAH increased more rapidly than that in patients taking PPZ. It was anticipated that ZAH, which was originally developed as a treatment for patients with Wilson’s disease with hypercupremia, would decrease serum Cu levels [32, 33]. However, in the present study, there was no significant decrease in serum Cu level until 24 weeks after the administration of 50 mg/day of ZAH for hypozincemia. Zn and Cu, which are absorbed through divalent metal transporter-1 (DMT1) on the intestinal epithelial cells, are known to antagonize the absorption of each other [34]. Previous studies have reported that Zn supplementation causes anemia, peripheral and optic neuropathy, and myelopathy when serum Cu is deficient [35, 36]. The Japanese Urology group demonstrated that serum Cu levels in the ZAH group were significantly lower than those in the PPZ group at 3 and 6 months after administration in patients under maintenance hemodialysis [24], despite using the same concentration of ZAH as that used in our study. Another study showed that when patients with cirrhosis complicated by hypozincemia were treated with ZAH (Zn content; 100 mg/day), the primary reason for the discontinuation of treatment was hypocupremia [37]. We speculated that Cu is reduced more in patients with impaired kidney function. It has been reported that a positive correlation exists between the serum Cu/Zn ratio and the salty taste recognition threshold or the awareness of dysgeusia [28]. In the present study, we investigated the Cu/Zn ratio and taste test results using not only salty taste but also four types of taste qualities. The mean Cu/Zn ratios before the administration of ZAH or PPZ were respectively 1.50 or 1.62, which were higher than the cut-off value of 1.1 for dysgeusia described by the previous study [28].

Taste evaluation after Zn replacement therapy was performed using the paper filter disk method. In the ZAH-administered group, a significant decrease in the taste threshold was observed at 12 weeks after administration, whereas in the PPZ-administered group, it was observed at 24 weeks. It was suggested that an increase in serum zinc concentration was associated with an improvement in taste. The administration of the zinc preparation decreased the threshold for salty and acidity among the four tastes but did not change the sweetness and bitterness. It has been reported that the serum Zn concentration was associated with salty taste recognition threshold [28]. It is also believed that the improvement in acidity is due to the reduction of tongue inflammation due to the anti-inflammatory effect of zinc preparations [13].

Finally, we compared the clinical parameters of patients in the improved and non-improved groups who received ZAH for 24 weeks. Our analyses revealed that the patients in the improved group were significantly younger than those in the non-improved group. The serum Zn concentration was higher in the improvement group than in the non-improved group. The Cu/Zn ratio was < 1.1 in 88% of patients in the improvement group compared with 56% in the non-improved group, which was significantly lower. The difference in the amount of Zn absorbed from the digestive tract between the two groups might have influenced the efficacy of ZAH. In the taste test, we evaluated four taste qualities and verified that the cut-off value for taste abnormality was 1.1. Based on the results, we believe that the Cu/Zn ratio cut-off value of 1.1 is reliable for dysgeusia. Previous reports showed that Zn can function as an anti-inflammatory agent in elderly subjects [13, 38]. As several patients with dysgeusia also suffer from glossitis, Zn administration can be expected to improve painful glossitis [39].

The present study was conducted at a single center and had a small sample size. In addition to zinc deficiency, other causes of taste disorders include pharmaceutical (chelating agents, vitamins and minerals), systemic (hypothyroidism, diabetes, neurological diseases such as Parkinson's and Alzheimer's), oral (Sjogren's syndrome and xerostomia) and psychogenic (clinically difficult to diagnose) causes. In addition, there is often more than one cause of the disorder in more than one case. In other words, although this study focused on patients with zinc deficiency taste disorder, it must be considered that other causes may be involved. Furthermore, ZAH and PPZ differ also in components other than Zn content. Thus, the present study has some limitations, and regional or selection bias might have affected the results. Prospective clinical studies with uniform patient backgrounds and drugs used should be conducted in the future.

## Conclusions

Serum Zn levels increased without reducing serum copper levels in patients with hypozincemia-induced dysgeusia treated with ZAH (50 mg/day) and taste improvement was observed in about 60% patients at 24 weeks of ZAH administration, suggesting Zn replacement using ZAH in patients with dysgeusia and lower serum Zn levels (< 80 µg/dL) might be considered as a new therapeutic approach.

## Data Availability

Data can be accessed on request, with relevant ethics approvals, by contacting the corresponding author.
